# Supplementary material for: Exploring Nitric Oxide as a Regulator in Salt Tolerance: Insights into Photosynthetic Efficiency in Maize
Source: Plants (Basel). 2024 May 10;13(10):1312. doi: 10.3390/plants13101312 (PMC11125177; doi:10.3390/plants13101312)
Supplement: Supplementary file 1 [file plants-13-01312-s001.zip › plants-2974020-supplementary.pdf]

Supplementary materials

Exploring Nitric Oxide as a Regulator in Salt Tolerance: Insights into Photosynthetic Efficiency in Maize

Georgi D. Rashkov, Martin A. Stefanov, Ekaterina K. Yotsova, Preslava B. Borisova, Anelia G. Dobrikova and Emilia L. Apostolova *

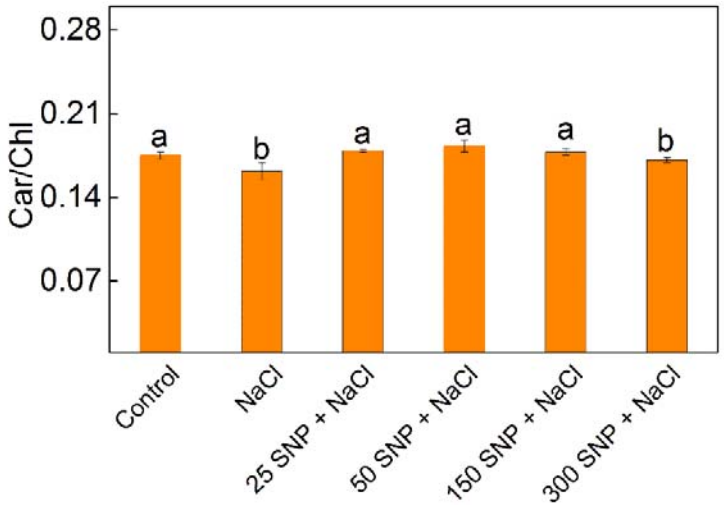

Figure S1. Impact of SNP on the total chlorophyll to carotenoid ratio of maize (*Zea mays* L. Kerala) under salt stress. The mean values (±SE) were calculated from 8 independent measurements. Significant differences between variants at $p < 0.05$ are marked by different letters

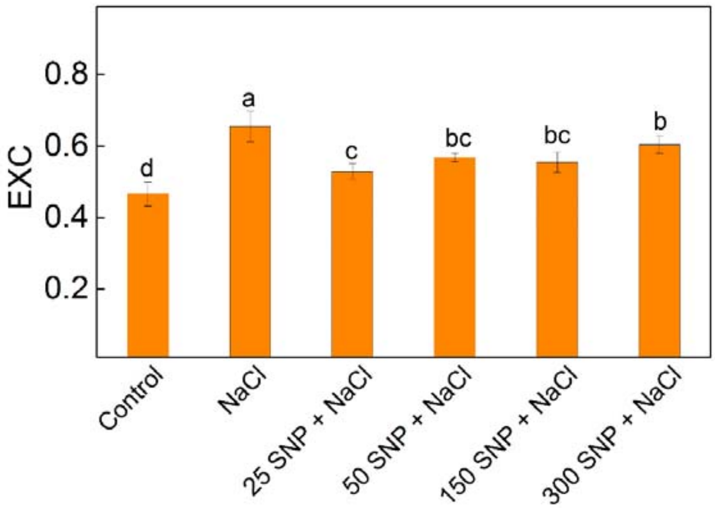

Figure S2. Effects of different SNP levels on excess excitation energy (EXC) in the leaves of one maize variety (*Zea mays* L. Kerala) during salt stress. We determined mean values (±SE) for 8 independent measurements. Significant differences between treatments at $p < 0.05$ are denoted by different letters.

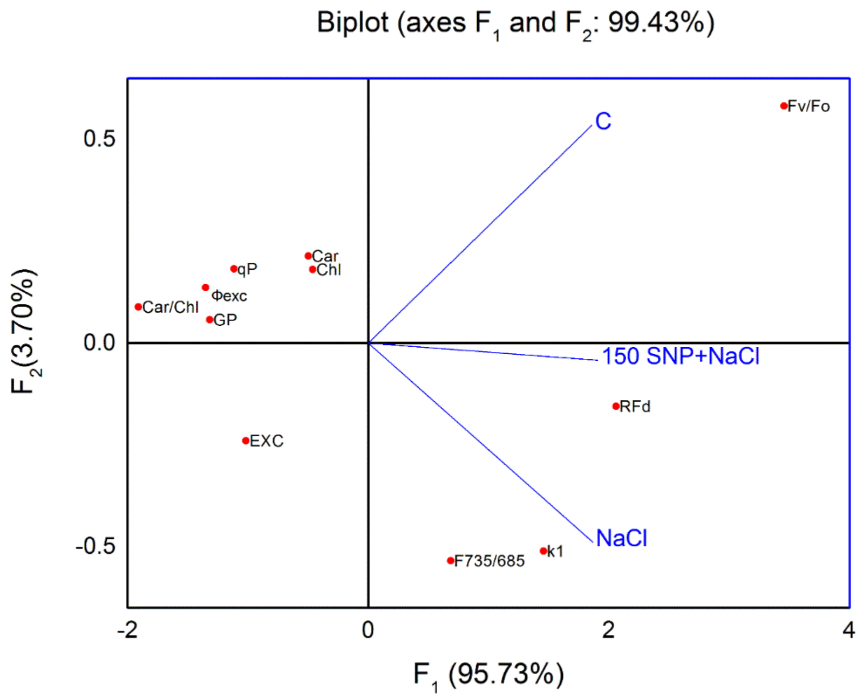

Figure S3. Principal component analysis (PCA) shows variation in the selected parameters after treatment with NaCl alone and co-treatment SNP and NaCl: PAM and 77K chlorophyll fluorescence, pigment content and thylakoid membrane fluidity.

Table S1. Variable contributions (loadings) for the principal component analysis model in Figure 1S.

Parameters	F1	F2
$F_{735/685}$	0.686	-0.535
Fv/Fo	3.456	0.582
R_{Fd}	2.060	-0.155
qP	-1.115	0.182
EXC	-1.015	-0.240
Car/Chl	-1.908	0.089
Chl	-0.459	0.181
Car	-0.498	0.214
k_1	1.459	-0.511
GP	-1.316	0.057
